# An Aluminum-Based Microfluidic Chip for Polymerase Chain Reaction Diagnosis

**DOI:** 10.3390/molecules28031085

**Published:** 2023-01-21

**Authors:** Siyu Yang, Ziyi Zhang, Qingyue Xian, Qi Song, Yiteng Liu, Yibo Gao, Weijia Wen

**Affiliations:** 1 Division of Emerging Interdisciplinary Areas, The Hong Kong University of Science and Technology, Clear Water Bay, Kowloon, Hong Kong SAR 999077, China; 2 Department of Physics, The Hong Kong University of Science and Technology, Clear Water Bay, Kowloon, Hong Kong SAR 999077, China; 3 Thrust of Advanced Materials, The Hong Kong University of Science and Technology (Guangzhou), Nansha, Guangzhou 511400, China; 4 HKUST Shenzhen-Hong Kong Collaborative Innovation Research Institute, Futian, Shenzhen 518000, China

**Keywords:** diagnosis, real-time polymerase chain reaction, nucleic acid diagnosis, aluminum chips, microfluidic chips, portability

## Abstract

Real-time polymerase chain reaction (real-time PCR) tests were successfully conducted in an aluminum-based microfluidic chip developed in this work. The reaction chamber was coated with silicone-modified epoxy resin to isolate the reaction system from metal surfaces, preventing the metal ions from interfering with the reaction process. The patterned aluminum substrate was bonded with a hydroxylated glass mask using silicone sealant at room temperature. The effect of thermal expansion was counteracted by the elasticity of cured silicone. With the heating process closely monitored, real-time PCR testing in reaction chambers proceeded smoothly, and the results show similar quantification cycle values to those of traditional test sets. Scanning electron microscope (SEM) and atomic force microscopy (AFM) images showed that the surface of the reaction chamber was smoothly coated, illustrating the promising coating and isolating properties. Energy-dispersive X-ray spectroscopy (EDS), X-ray photoelectron spectroscopy (XPS), and inductively coupled plasma-optical emission spectrometer (ICP-OES) showed that no metal ions escaped from the metal to the chip surface. Fourier-transform infrared spectroscopy (FTIR) was used to check the surface chemical state before and after tests, and the unchanged infrared absorption peaks indicated the unreacted, antifouling surface. The limit of detection (LOD) of at least two copies can be obtained in this chip.

## 1. Introduction

A polymerase chain reaction (PCR) is a fundamental technique for molecular biological tests invented by Mullis [1]. It can amplify deoxyribonucleic acid (DNA) molecules or their fragments with specific sequences in vitro and further detect certain amplicons [2,3,4]. It has had a profound impact on molecular biology and revolutionized DNA profiling research and the synthesis of nucleic acid molecules. Since the late 20th century, PCR and its derivative techniques have been extensively used in genetics [5,6,7], cloning research [8], and forensic science [9,10] due to their specificity and accuracy.

Higuchi et al. [11], in their study, integrated fluorescent molecules (Ethidium bromide (EtBr)) with PCR that can monitor the amplifying process in situ by a video camera and for the subsequent development of quantitative PCR (qPCR or real-time PCR). Heid et al. [12] successfully conducted qPCR tests in a closed tube system with dual-labeled fluorogenic probes in 1996, which became a standard molecular method for molecular biology. 

A real-time PCR process involves 40–45 repetitive heating cycles, including two or three temperature stages for each cycle. DNA samples are denatured at 90–95 °C in the first stage, and the system is then decreased to 55–75 °C to anneal the primer with fluorophore ends. DNA samples are doubled in the last stage at 72–78 °C for each cycle [13]. In a general analysis, the second and third stages can be consolidated into one stage at similar temperatures, with the ideal range being 50–60 °C [14]. The fluorescent signal increases exponentially with the fluorophores released into the system during amplification, while the intensity of relative fluorescent units (RFU) is recorded in real time. When the signal crosses a certain threshold, the quantitative cycle (Cq) values are read out as quantitative references [15]. Real-time PCR provides both qualitative and quantitative results within its instant data output, making it widely used in research and practical applications, especially in recent years for diagnosing diseases such as cancer, SARS-Cov-2, monkeypox, and Ebola [16,17,18,19,20,21]. It has also been used for environmental research, husbandry testing, and food safety [22,23]. 

Metal heating plates and polypropylene (PP) tubes are typically employed in conventional real-time PCR testing, resulting in deficiencies such as slow heating and cooling rates, as well as high sample and reagent consumption [24]. The process is accompanied by nearly 100 ramp-ups and ramp-downs that usually take hours. Wilding et al. [25] realized PCR in a silicon substrate in 1994, which constituted a milestone in microfluidic usage for PCR. Kopp et al. [26] developed the first microfluidic chips for continuous-flow PCR that significantly reduced the time consumption of the process. Since the beginning of the 21st century, the development of micro-electro-mechanical-system (MEMS) and microfluidic techniques has led to the extensive use of microchips in genetic analysis and DNA diagnosis. The smaller scale and organized flow channels and chambers improve the surface-to-volume ratio, resulting in the large energy exchange area [27]. 

Furthermore, the fast heat and mass transfer of microfluidic chips relative to conventional PCR tubes enables more rapid and efficient PCR tests [28]. The advantages of microfluidic chips, such as high throughput, disposability, portability, low cost, and reduced reagent and sample consumption, make microfluidic chips an ideal method for daily clinical analysis [29,30,31]. Nevertheless, their potential for point-of-care medical diagnostic solutions, food safety, and environmental monitoring has compelled researchers to design system patterns for various applications and integrations in recent years [32,33,34].

These intrinsic advantages of microfluidic PCR are well replicated in real-time PCR tests on chips, opening access to a powerful approach for detecting multiple targets simultaneously with high resolution [35]. Silicon and transparent polymers such as PDMS and PMMA are the most used array substrates. The patterned mold can be fabricated with photolithography or a computer numeric controlled (CNC) machine for polymer-based chips, while the dry-etching technique is necessary for silicon-based chips [36]. Due to the relatively low thermal conductivity and stability of polymers, silicon-based chips remain the top choice for medical diagnostic applications, even though they are more expensive to produce than chips made of polymers. Since 2012, we have reported several self-designed microfluidic chips and a detection system for on-chip real-time PCR integrated with silicon-based microheaters, realizing high-efficiency tests within 25 min [37,38]. 

Although microfluidic real-time PCR tests are considered an effective and efficient method for clinical analysis, unlike conventional devices, their application in the daily diagnosis of viruses is hampered by capacity concerns and expensive production processes, especially in silicon consumable manufacture and costs. Additionally, the capacity of silicon-based chips is difficult to guarantee if a large number of samples and tests are needed in the short term. Therefore, there is an urgent need to develop a low-cost and mass-producible microfluidic chip to fill this gap in research and industry. To overcome this, suitable materials for silicon replacement should be identified. Plastic is also one of the most common materials in diagnostic applications. However, it always has a much lower thermal conductivity than the metal substrate. For example, the most commonly used polymer, polypropylene (PP), only has a conductivity of around 0.22 W/m•K, even in a high density [39]. In contrast, aluminum and its alloy have a conductivity of 251 W/m•K, increasing as the temperature rises, which is more significant than normal plastic [40]. So far, there has been little research into developing microfluidic chips for PCR using materials other than silicon wafers and PDMS. Metal is considered to have good ductility and thermal conductivity, higher than monocrystalline silicon. However, since metal ions can interfere with the necessary components of nucleic acid detection reagents, there is a general concern that they cannot be used as the substrate for biological analysis. 

In this study, the use of aluminum chips in nucleic acid detection can be achieved by employing suitable isolation materials that can prevent metal ions from escaping from the chip surface. Epoxy resin is a commonly used coating material for isolation with excellent adhesive properties and biocompatibility. Furthermore, the cured epoxy resin coating layer has a superior bonding strength with aluminum surfaces of up to 9 MPa. It does not change with the temperature or external contact medium, making it suitable to be the coating material in this study [41,42]. Recently, Cheng et al. [43] developed a rigid microfluidic chip using epoxy resin casting and realized efficient PCR on the chip, indicating its compatibility with PCR. Moreover, silicone modification can further enhance the mechanical property and thermal stability of epoxy resin, making it ideal for chemical and solvent isolation to protect our reaction system from being affected by metal [44]. 

An aluminum-based microfluidic chip for real-time PCR diagnosis with the designed pattern was developed. The pattern was manufactured by CNC, which is one of the most common methods in the field of manufacturing. It has been applied in many existing studies for mass production, such as flat patterns, microarrays, and electrodes [45,46,47]. Silicone-modified epoxy resin was coated evenly on the chip’s surface to isolate the reaction system from the metal surface, which prevented metal ions from diffusing and degrading the PCR reaction system. Low-ion glass masks for fluorescent detection were bonded with the chip with silicone sealant using the screen-printing technique. Real-time PCR in silicon-based PCR chips and traditional polypropylene tube strips was used as the standard reference. The real-time PCR amplification result from the described chips showed no differences when compared with data from standard groups. All the materials for fabricating chips are readily available. The inexpensive materials and manufacturing can significantly reduce costs, and the high thermal conductivity of metals can improve reaction efficiency. Additionally, its room-temperature production process and portability facilitate the development of reusable, environmentally friendly, and low-cost portable nucleic acid diagnostic equipment. The described chips will be of great use in the future for public health monitoring and intelligent home self-diagnosis for many common diseases.

## 2. Results and Discussion

Several works and pattern designs for nucleic acid amplification tests of silicon-based chips have been reported in our research in recent years [37,38,48]. When engraving the pattern on the aluminum substrate, the channel arrangement was not changed as compared with our previous study, but the ratio of the inlet and outlet channels was adjusted to allow for a more straightforward spraying process later. The width of inlets and outlets was expanded to 0.5 mm, while the depth remained unchanged at 0.3 mm. The wider inlet and outlet channels enable smoother gas and liquid flow and contribute to protein immobilization as a potential application. 

**Scheme 1:**
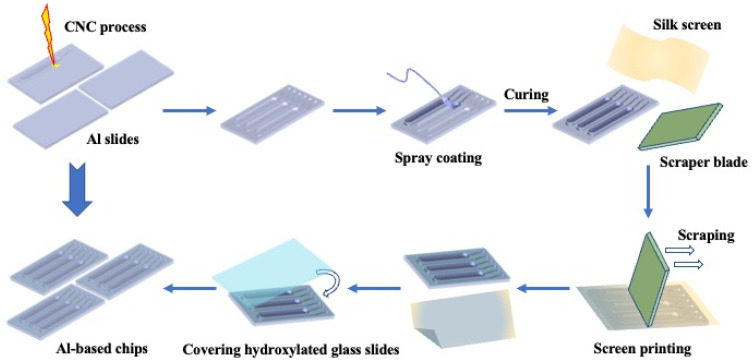
Schematic representation of process flow for the aluminum-based chips.

Epoxy resin has been proven to have promising biocompatibility and is used in biological experiments as a substrate or coating material. The coating layer was controlled to 20 μm, which allowed for the separation of the reaction system while preventing blockage. Acetone, a typical solvent for dissolving and diluting epoxy resin, was used to regulate the coating material’s viscosity. The thickness of the coated layer is controlled by measuring the weight of the liquid sprayed onto the chips. The natural silicone sealant used in this study had very low fluidity; therefore, we did not require patterns on the screen during the printing process. After the scraper swept past the surface of the chip, only the convex surface of the chip was covered with the sealant, and the reaction chambers and flow channels were not affected (Scheme 1). Mineral oil was added to adjust the fluidity and viscosity of the sealant before printing. Nevertheless, the elasticity of cured natural silicone can counteract the effect of the thermal expansion difference between the chip substrate and the covering mask. Bonded chips were cured at room temperature, without high-pressure or extreme conditions. The antifouling property of silicone-modified epoxy resin further realizes a low protein absorption and easy recleaning process, realizing the reusability of the aluminum-based chips [49,50]. It is 85% cheaper for batch production than silicon-based chips, which is beneficial for the practical application of this chip.

### 2.1. Surface Morphology of the Coated Area of Chips

**Figure 1:**
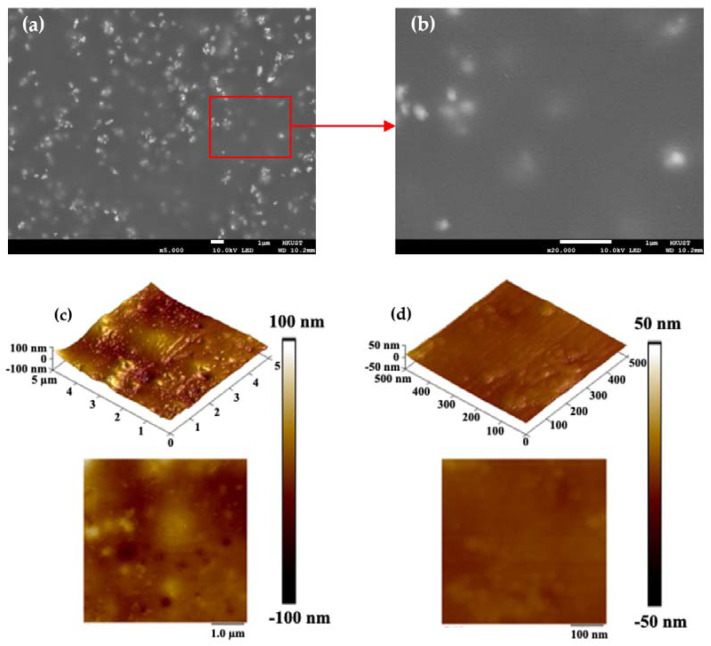
(**a**,**b**) The ×5000 and ×20,000 SEM images, respectively, for the coated reaction chamber surface of aluminum-based chip; (**c**,**d**) 3D and 2D tapping-mode AFM images of the coated chip surface from a 5 µm × 5 µm area and a 500 nm × 500 nm area, respectively.

According to our previous findings, metal ions could affect the function of the active ingredients, such as Taq polymerase and fluorescent primers, when the concentration reaches a certain threshold [51]. In this case, one of the critical points for running nucleic acid amplifications in the described chips is to isolate the metal surface from the reaction system, where the coating material plays a role. The SEM images in Figure 1 show the surface morphology of the coated chips, indicating the uniformly coated surface of the reaction chamber. No apparent cracks or micropores were found in the covering layer. Some nanoscale cracks observed on the surface are caused by the thermionic emission of electrons from SEM. White granular traces in the coating layer are evenly distributed. As is well-known, the epoxy resin phase and silicone phase were originally incompatible, and compatibilizers realized the modification. Therefore, it can be inferred that silicone particles cause the aforementioned phenomenon in the resin, which will not affect the surface biocompatibility, uniformity, and subsequent PCR tests. Several earlier reports have established this [44,52,53].

Functional biomolecules, including proteins, could absorb on the rough surface, which would hinder the function of the enzyme and the amplification of the nucleic acid [54]. Meanwhile, a smooth covering layer guarantees the sequestration of metal ions and homogeneous thermal conductivity. Therefore, surface roughness must be ensured for the PCR application of this chip. Figure 1c and Figure 1d show the 3D and 2D tapping-mode AFM images. The height difference of the covering layer was less than 200 nm, and a height difference of less than 100 nm was obtained within the 500 nm × 500 nm area. Some silicone particles can be identified in the image. No cracks or micropores were found in the covering layer either. This further proved the excellent sequestration of metal ions, consistent with the following element analysis and PCR result. 

### 2.2. Surface Elemental Analysis of the Chips

**Figure 2:**
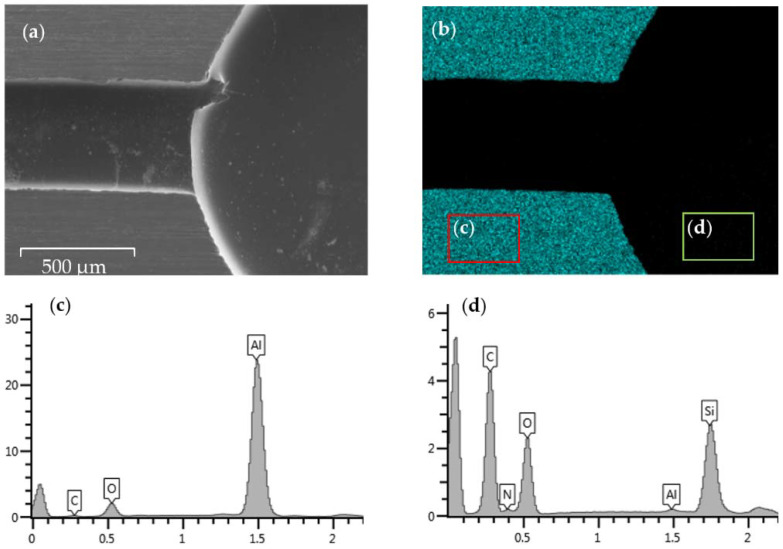
(**a**) SEM image of the tested area for EDS mapping. (**b**) EDS mapping result for Al element on the tested site; two areas framed in different colors will be plotted in the map sum spectrums. (**c**) Map sum spectrum for the convex surface of the chip, red frame in Figure 2b. (**d**) Map sum spectrums for the coated surface of the chip, green frame in Figure 2b.

**Figure 3:**
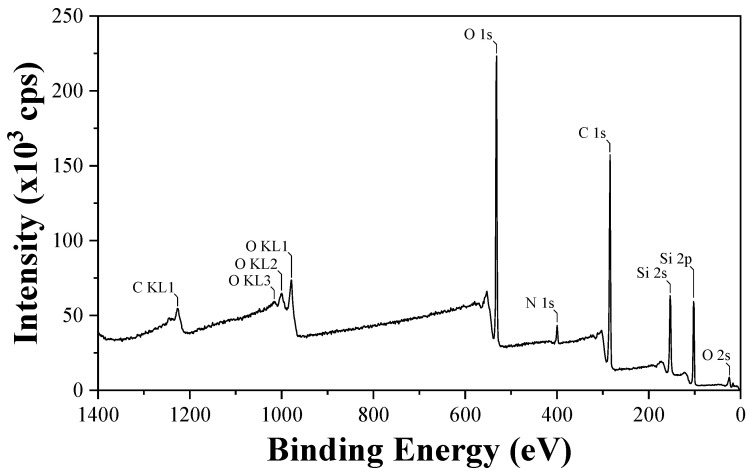
XPS spectrum of the coated aluminum-based chip surface.

SEM-EDS was used to investigate the elemental composition of the surface at a more microscopic level. For comparison, an aluminum chip without any coatings is also characterized. Figure 2a,b shows the SEM image for the tested area and the EDS mapping result for the Al element in this area, respectively. Only the uncoated convex surface can be observed to be covered with aluminum elements. The reaction chamber and flow channel missing Al signals can be easily identified. Two areas are framed to conduct a more detailed mapping analysis. One area is from the convex surface that is supposed to be bare metal, while the other is from the coated surface of the chip. The mapping analysis for the bare aluminum chips as a reference is also conducted, obtaining the map sum spectrum in Figure S1. The map sum spectrums for the two framed areas are shown in Figure 2c,d, while Table 1 shows the proportion of each element on the chamber surface as determined by each spectrum. When comparing the compositions of the bare chip and the coated surface, the Al mass percentage is reduced from 89% to 0.26%, and the atomic percentage from 79.74% to 0.14%. The percentages of Carbon (C), nitrogen (N), oxygen (O), and silicon (Si) that are supposed to be contained in silicone-modified epoxy resin and its curing agents and compatibilizers increase and dominate the surface element content instead, indicating excellent insulation of the metal elements. The small amount of Al element on the coated surface is deduced to be caused by the polishing and coating process before SEM. The energy peak of the platinum coated on the chip before SEM and SEM-EDS also appears in the spectrum and is not considered. From the mapping result of the convex surface of the coated chip, no apparent changes were found, except the increase in the proportion of the oxygen content. This phenomenon is attributed to the heating process during fabrication, which can promote the oxidation of aluminum surfaces. XPS analysis (Figure 3) for the coated surface further demonstrates the presence of O 2s, Si 2p, Si 2s, C 1s, N 1s, O 1s, O KL1, O KL2, O KL3, and C KL1. This is in line with the result mentioned earlier.

### 2.3. Thermodynamic Characterization of Coated Aluminum Chips

**Figure 4:**
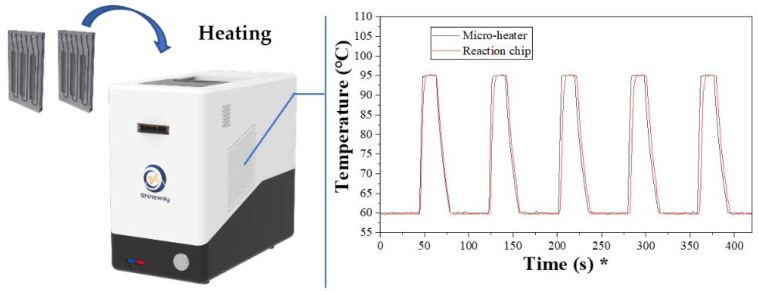
Schematic diagram of the heating process and temperature changes of the microheater and the aluminum chip in five randomly selected cycles during the real-time PCR process. * The first point in the randomly selected thermal cycles is defined as 0 s.

During the real-time PCR running, the temperature changes of the microheater previously developed were monitored by the built-in sensor, and an external sensor detected the chip sample temperature. Five cycles were randomly chosen as representatives, and the temperature profile for the microheater and the chip are plotted in Figure 4. For the temperature profile of the microheater, it only takes 5 s for the heater to increase from 60 °C to 95 °C, and the cooling process takes 20 s. This is in line with the report of our previous research. As regards the profile of the aluminum chip, only a 1.5 s delay, caused by the conduction of heat between the heater surface and chip surface, was observed during the heating process. This is within expectations and is acceptable. In the described chips, the 45 cycles of a real-time PCR process can be completed in 63 min. On the other hand, conventional tests in Control B, which ran the reaction in traditional polypropylene tube strips, took about 90 min to finish the process and were greatly optimized by this work.

### 2.4. Real-Time PCR Tests in the Aluminum-Based Chips

Determining if the newly designed chips can be used in our self-built real-time system with the silicon microheater is crucial for this study. From this point, experiments group (Control A). A real-time PCR mix, as prepared, was injected into reaction chambers through inlet channels. Control A chips were tested simultaneously. The compatibility and function of the silicon-based chips used in Control A were thoroughly tested before [48,55]. The quantification cycle (Cq) values were considered for evaluating the function and efficiency of the test in the present study. 

**Figure 5:**
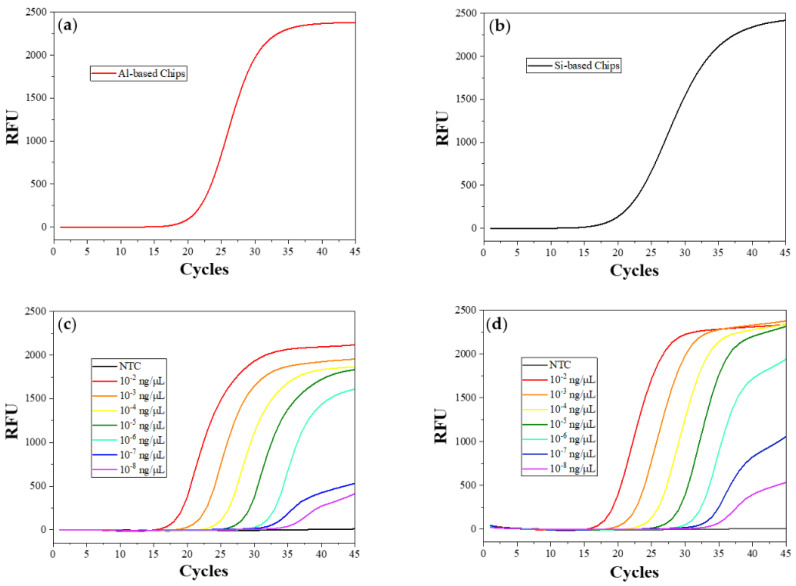
Amplification result of (**a**) the real-time PCR test in the aluminum-based chip, (**b**) the real-time PCR test in the silicon-based chip (Control A), (**c**) the real-time PCR tests in the aluminum-based chips testing 10^−2^ ng/reaction (about 2.5 × 10^6^ copies) to 10^−8^ ng/reaction (about 2 copies); (**d**) the real-time PCR tests in traditional polypropylene tubes (Control B) testing 10^−2^ ng/reaction (about 2.5 × 10^6^ copies) to 10^−8^ ng/reaction (about 2 copies).

The amplification result of the real-time PCR test using the described chips when it tests 10^−3^ ng HBV templates (about 2.5 × 10^5^ copies in total) is presented in Figure 5a, and that of Control A is shown in Figure 5b. The real-time fluorescent images from the first to the last cycle of reactions in aluminum-based chips are shown in Figure S2, which corresponds with its amplification curve. No significant differences can be observed between the Cq values of the test in aluminum-based chips and silicon-based chips from the amplification curves, indicating the usability of this aluminum chip in the real-time system with the silicon microheater we manufactured [37]. The curve shapes are similar, with smooth S-shaped amplification curves, except for the unidentical slopes of the plateau stage at the last five cycles, which do not affect the judgment of test results. The substrate and the mask we select in this work have a low coefficient of thermal expansion. The effect caused by the thermal expansion of the two components can be counteracted by the cured silicone, as mentioned before. In this case, neither reaction-mix leakage nor solvent evaporation was found when the mix was heated in the chips described, proving our inference. Some bubbles found in the fluorescent images were caused by air trapped on the chip surface and the adhesive layer when the reaction mix was injected into the chamber. It does not affect the accuracy of the result if we select a certain detection area and optimize the result with our PCR analyzer [56].

To determine the limit of detection (LOD) of the described chips, we set a gradient of template concentration from 10^−2^ ng/reaction (about 2.5 × 10^6^ copies) to 10^−8^ ng/reaction (about two copies in total) for tests in aluminum-based chips and Control B. The amplification curves of each test are shown in Figure 5c,d, respectively. The same test was repeated 5 times in each of the same chips, and the mean Cq values of amplification curves for testing different template concentrations in all reaction units were read out and are shown in Table 2. All sets of data have standard deviations within 0.5, which is acceptable. There is no significant difference between the amplification Cq value in aluminum-based chips and that in PP tubes when testing more than 10^−5^ ng templates (around 2500 copies). A Cq value delay of 1.3 to 1.6 cycles appears when the chips test fewer than 10^−6^ ng of templates (about 250 copies). However, the amplification can still show a significant increase in fluorescence within 40 cycles, which can satisfy the testing requirement and is acceptable in this study. This phenomenon also agrees with the conclusion that the amplification is more likely to be affected by the reaction system and heating properties when the template concentration is low [57]. Since the curved shapes of the plateau stage of the last five cycles mainly depend on the detection equipment itself, the Cq value differences are the only factor considered in the analysis. This result demonstrates that this chip is fully compatible with the microfluidic chip-based system we developed [48,58]. It has the potential for us to find more compatible devices and test technology in the future.

### 2.5. The Chemical State of the Chip Surface

**Figure 6:**
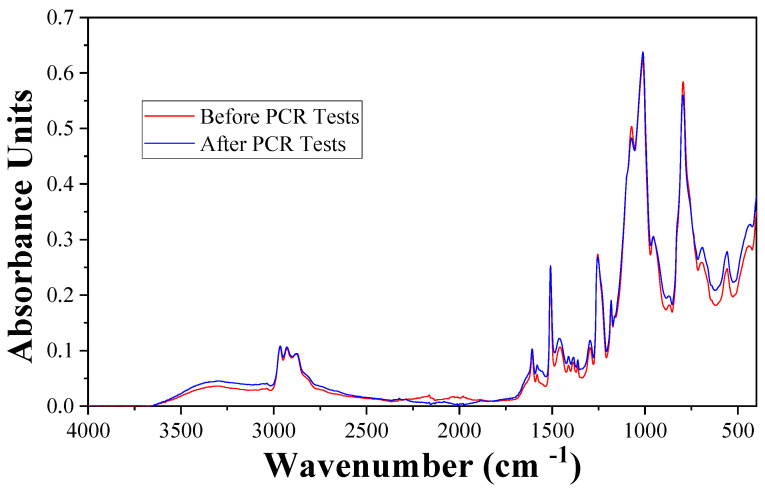
FTIR spectrum of the coated aluminum-based chip surface before and after running a real-time PCR test.

To ensure that the reaction proceeds normally, the coated surface should not interreact with the PCR reaction system. FTIR analyses were conducted twice for the same chip before and after running the PCR. The spectrum is shown in Figure 6. These two spectrums are highly coincident. Both spectrums show major absorptions at 2925 cm^−1^ (CH2), 2877 cm^−1^ (CH3), 2966 cm^−1^, 1255 cm^−1^, 804 cm^−1^ (Si-CH3), 1606 cm^−1^, 1508 cm^−1^, 1460 cm^−1^ (phenyl), 1010 cm^−1^ (Si-O-Si), and 915 cm^−1^ (epoxy group). This means there is no apparent discrepancy between the surface chemical state before and after the PCR test. The ingredients in the PCR reaction system neither react with the coating material nor absorb on the chip surface, which is consistent with the unchanged PCR result.

### 2.6. Metal Ions Escape Analysis (ICP-OES)

Based on the characterization of the surface of the chip, we analyzed the metal ion concentration in the reaction mix. Solutions prepared as mentioned, containing reaction mix before reactions (Solution 1), reaction mix after reacting in coated Al-chips and provided successful amplifications (Solution 2), and reaction mix after processing in un-coated Al-chips (Solution 3) that did not show any amplification signal (data is not shown), were analyzed for metal concentration with ICP-OES simultaneously. Aside from aluminum, aluminum alloy 6061 has a probability of containing magnesium (Mg), silicon (Si), iron (Fe), copper (Cu), chromium (Cr), zinc (Zn), and manganese (Mn). Among these elements, Mg and Si will not affect the PCR process according to our work, and others were set as the targets for us to detect.

The results after calculation are shown in Table 3. For the reaction mix in untreated chips, 3.23 nmol Al ions were solved in the system at the end of the reaction, even higher than the Mg ions we added for enzyme activation during preparation. Other metals had also increased by varying degrees, explaining the failure amplification. In contrast, the reaction mix from the designed chip prevents most of the metal ions from escaping from the metal surface to the reaction mix during the heating process. A slight increase in the concentration of Al and Cu may be caused by a coating defect in one or two chambers or by systematic errors. However, considering the unaffected PCR result and the low trend of increase, this can be ignored. This excellent isolation result agrees with the element analysis and the real-time PCR result.

## 3. Materials and Methods

### 3.1. Materials

Aluminum alloy 6061, with a low thermal expansion coefficient and no heavy metals, was chosen as the substrate of the chips. The silicone-modified epoxy resin, ALBIDUR EP 2240 (Evonik), was purchased from Alibaba. The silicone sealant used in this study, GlassSil-Neutral by GE Silicones (Japan), was purchased from Momentive Performance Materials. Low-ion glass slides were customized by Guluo Glass (China). The 420-mesh silkscreens used for the printing technique were obtained from NBC Meshtec (Japan). 

Acetone, isopropanol, and ethanol were purchased from Sigma-Aldrich. All raw materials were used as received. 

The basic real-time PCR reaction system was prepared following our previous research [59]. Potassium chloride, Trizma^®^ base, hydrochloric acid (36.5–38.0%), magnesium chloride, Tween 20, PCR-grade water, and Axygen^®^ microplate aluminum sealing film were purchased from Sigma-Aldrich. Deoxyribonucleotide triphosphates (dNTPs) in the 25 mM solution were supplied by ThermoFisher Scientific. The template we used is the Hepatitis B virus (HBV) plasmids synthesized by GenScript Biotech, a standard template for verifying PCR tests and material availability. Primers and TaqMan probes customized for the HBV plasmids were also supplied by GenScript Biotech. A 6-carboxy fluorescein (6-FAM) was used as a fluorophore, and black hole quencher 1 (BHQ-1) was used as a quencher (Table S1). All the amplified sequences are the same as in our report of a previous study. Taq polymerase was purchased from Ampliqon A/S. Potassium chloride and magnesium chloride were prepared into 1 M and 0.05 M solutions in PCR-grade water, respectively. Trizma^®^ base and hydrochloric acid were prepared as 1M Tris-HCl buffer solutions at pH 8.09 in PCR-grade water for subsequent use. Primers and probes were merged into a 100 µM mix (p-mix) in PCR-grade water for later use. All materials were prepared in a biosafety laboratory.

### 3.2. Fabrication of Aluminum-Based Chips

Scheme 1 shows the fabrication procedure of the sealed aluminum chips for the PCR test. The aluminum chips with three reaction chambers were processed by CNC with MIKRON-VCP600 Machining Centre. Silicone-modified epoxy resin was diluted with acetone to reduce the viscosity. The covering layer was directly spray-coated on the surface of the reacting chambers and flow channels by air pressure and a micro nozzle. The coated chips were held at room temperature for 8 h for curing and then transferred to 80 ℃ for post-curing for 24 h. Before bonding with the low-ion glass mask, the coated chips were washed with ethanol, acetone, and isopropanol in sequence to remove unreacted chemicals from the surface. The chips were held at 80 °C for another 1 h to evaporate the remaining solvent. Thereafter, the natural silicone sealant was printed on the convex surface using screen printing technology with a 420-mesh silk screen. Subsequently, low-ion glass masks with high light transmittance were hydroxylated by air plasma with a plasma cleaner (Harrick Plasma, PDC-002) and immediately brought to conformal contact with patterned aluminum chips. The bonded chips were cured at normal pressure and room temperature for 24 h and directly used for real-time PCR tests. 

### 3.3. Surface Morphology, Elemental Characterization, and Chemical State of the Chip Surface

The surface morphology of the coated aluminum chips was examined using a scanning electron microscope (SEM, JEOL-7100), and surface element analysis was conducted with the in-build energy-dispersive X-ray spectroscopy system in the SEM (SEM-EDS, JEOL-7100). The surface roughness of the chips was characterized using atomic force microscopy (AFM) imaging (Dimension Icon). The elemental composition of the coated chip’s surface was also identified using X-ray photoelectron spectroscopy (XPS) with an Axis Ultra DLD X-ray photoelectron spectrometer (Kratos). Attenuated total reflection-Fourier-transform infrared spectroscopy (ATR-FTIR) for the surface of the chip was conducted with Vertex 70 Hyperion 1000 (Bruker). 

### 3.4. Heating Properties Monitoring

In addition to the reaction substrate, the temperature is another crucial point for successful reactions. The self-built SWM-02 real-time PCR system has an in-build temperature monitor to control the temperature of microheaters and reaction chips. The mechanical structure, heating, and detection principle were interpreted in our previous report [55]. During the PCR process, the temperature of the micro-heater was monitored by the built-in temperature sensor, and an external temperature sensor detected the temperature of the reaction chip.

### 3.5. Real-Time PCR Test in Aluminum-Based Chips

The whole PCR reaction mix required in one reaction chamber was defined as one reaction unit. To each reaction unit, 0.5 μL 1 M KCl, 0.5 μL 1 M Tris−HCl (pH 8.09), 0.10 μL of 1 M (NH4)_2_ SO_4_, 0.04 μL of 0.05 M MgCl_2_, 0.16 μL of 25 mM dNTPs, 2 units Taq polymerase, 0.04 μL of 100 mM p-mix, and corresponding templates were added. Each reaction unit was diluted with PCR-grade water to a final volume of 10 μL and pipetted into reaction chambers. Chip inlets and outlets were sealed with aluminum sealing film. A set of reactions processed in silicon-based PCR chips (Control A), developed in our previous study, and another set of traditional reaction polypropylene tube strips (Control B) were used as the reference. The reactions in the aluminum chips and Control A were performed simultaneously in the SWM-02 real-time PCR system for PCR chips (Shineway), and the reactions in Control B were performed in the CFX96 Touch real-time PCR detection system (Bio-rad). All reactions were conducted with 45 heating cycles, as described earlier.

### 3.6. Metal Ions Escape Analysis

After running the PCR process in the coated aluminum chips and obtaining amplification data, the reaction mix was pipetted out and collected. Afterward, the mixture was diluted with DI water to form a 1 unit/mL solution. The same procedure was conducted for a group of reactions in uncoated aluminum chips. Another solution with the same concentration but an unreacted PCR mix was prepared as a reference. All three sets of solution were analyzed using an inductively coupled plasma-optical emission spectrometer (ICP-OES, Perkin Elmer-Avio 200). In this case, the amount of metal ions that escape to the reaction mix can be calculated using the following equation,

$$Esc=\frac{C}{M},$$

where *Esc* is the number of escaped metal ions in each reaction unit (nmol/unit), *C* is the concentration of metal ions of the tested solution in ICP-OES (ppb), and *M* is the molar mass of the corresponding metal ion (g/mol).

## 4. Conclusions

An aluminum-based microfluidic chip for real-time PCR was developed in this study. Aluminum alloy 6061 sheets are engraved using CNC with reaction patterns and coated with silicone-modified epoxy resin. Neutral silicone sealant bondeds glass slides and the aluminum substrate while offsetting the thermal expansion effect. The preparation process was conducted at room temperature with inexpensive and readily available materials. The covering layer was smoothly coated in the reaction chamber with a height difference of less than 200 nm. ICP-OES analysis shows metal ions that can affect the reaction system are well prevented from escaping from the metal surface to the reaction system. Only a 1.5 s temperature delay between the reaction chips and the microheater was found due to heat conduction. Real-time PCR tests were successfully conducted in the chips, resulting in excellent amplification curves and higher test efficiency. As a result, the standard reaction can be completed in 63 min, which is highly optimized in contrast with the reaction in conventional PP tube strips. The LOD of two copies of templates was reached in the described chips. No interactions between the reaction system and the reaction chamber were observed. 

Consequently, the aluminum-based chip developed in this work can be applied for daily nucleic acid tests and diagnosis. Furthermore, the manufacturing procedure eliminates the complex etching process of the silicon-based chip with the benefit of being both cost-effective and environmentally friendly. The fabrication process was conducted at room temperature; no high pressure or extreme conditions were required, making it suitable for mass production. When we use CNC to manufacture the reacting chips instead of photolithography and dry etching, the production expenditures can be reduced by 60% in a typical lab application and reduced by 85% for mass production. Integrating with the portable testing device we developed, it has the potential for practical home-use applications and significantly benefits point-of-care testing for food safety, environmental research, and many common diseases such as HBV, COVID-19, and monkeypox.

## Figures and Tables

**Table 1:** The element content of the uncoated and coated chip surface.

	Uncoated Chip		Coated Chip (Convex Surface)		Coated Chip (Coated Surface)	
**Element**	Wt %	Atomic %	Wt %	Atomic %	Wt %	Atomic %
**C**	8.34	16.72	7.71	14.35	61.04	70.48
**N**	0	0	0	0	0.09	0.09
**O**	2.35	3.54	16.07	22.47	27.41	23.76
**A** **l**	89.31	79.74	76.22	63.18	0.26	0.14
**Si**	0	0	0	0	11.20	5.53
**Total**	100.00	100.00	100.00	100.00	100.00	100.00

**Table 2:** Cq values of real-time PCR tests with different template concentrations in aluminum-based chips and control B.

Template (ng/Reaction)	Al-Based Chips (Cq)	PP Tubes (Cq)
**NTC**	N/A	N/A
**10^−2^**	18.74	19.07
**10^−3^**	22.23	22.51
**10^−4^**	26.04	25.83
**10^−5^**	29.39	29.03
**10^−6^**	33.45	32.18
**10^−7^**	35.51	34.76
**10^−8^**	38.88	37.34

**Table 3:** Metal concentration (nmol/unit) of the PCR mix before and after reaction process *.

	Al (nmol/Unit)	Fe (nmol/Unit)	Cu (nmol/Unit)	Cr (nmol/Unit)	Zn (nmol/Unit)	Mn (nmol/Unit)
**S** **olution 1**	0	0	0	0	0	0
**S** **olution 2**	0.01	0	0.01	0	0	0
**S** **olution 3**	3.23	0.02	0.04	0.01	0	0.01

* Solution 1: original mix; Solution 2: mix processed in coated chips; Solution 3: mix processed in uncoated chips.

## Data Availability

Not applicable.

## Supplementary Materials

The following supporting information can be downloaded at: https://www.mdpi.com/article/10.3390/molecules28031085/s1, Table S1: Information of primers and probes used in this study; Figure S1: EDS map sum spectrum for the surface of chips before coating with silicone-modified epoxy resin; Figure S2: Fluorescent images from each cycle of the test in the aluminum-based chip.

## References

[1] Saiki R., Scharf S., Faloona F., Mullis K., Horn G., Erlich H., Arnheim N. (1985). Enzymatic amplification of beta-globin genomic sequences and restriction site analysis for diagnosis of sickle cell anemia. Science.

[2] Mullis K., Faloona F., Scharf S., Saiki R., Horn G., Erlich H. (1986). Specific enzymatic amplification of DNA in vitro: The polymerase chain reaction. Proceedings of the Cold Spring Harbor Symposia on Quantitative Biology.

[3] Saiki R.K., Bugawan T.L., Horn G.T., Mullis K.B., Erlich H.A. (1986). Analysis of enzymatically amplified β-globin and HLA-DQα DNA with allele-specific oligonucleotide probes. Nature.

[4] Saiki R., Gelfand D., Stoffel S., Scharf S., Higuchi R., Horn G., Mullis K., Erlich H. (1988). Primer-directed enzymatic amplification of DNA with a thermostable DNA polymerase. Science.

[5] Welsh J., Petersen C., McClelland M. (1991). Polymorphisms generated by arbitrarily primed PCR in the mouse: Application to strain identification and genetic mapping. Nucleic Acids Res..

[6] Li J., Wang L., Mamon H., Kulke M.H., Berbeco R., Makrigiorgos G.M. (2008). Replacing PCR with COLD-PCR enriches variant DNA sequences and redefines the sensitivity of genetic testing. Nat. Med..

[7] Hoshika S., Chen F., Leal N.A., Benner S.A. (2010). Artificial Genetic Systems: Self-Avoiding DNA in PCR and Multiplexed PCR. Angew. Chem..

[8] Schaefer B.C. (1995). Revolutions in rapid amplification of cDNA ends: New strategies for polymerase chain reaction cloning of full-length cDNA ends. Anal. Biochem..

[9] Jobling M.A., Gill P. (2004). Encoded evidence: DNA in forensic analysis. Nat. Rev. Genet..

[10] Huang Y., Chen X., Li X., Shu P., Wang H., Hou T., Wang Y., Song F., Zhang J. (2022). A proof-of-principle study on implementing polymerase chain displacement reaction (PCDR) to improve forensic low-template DNA analysis. Forensic Sci. Int. Genet..

[11] Higuchi R., Fockler C., Dollinger G., Watson R. (1993). Kinetic PCR Analysis: Real-time Monitoring of DNA Amplification Reactions. Nat. Biotechnol..

[12] Heid C.A., Stevens J., Livak K.J., Williams P.M. (1996). Real time quantitative PCR. Genome Res..

[13] Mackay I.M., Arden K.E., Nitsche A. (2002). Real-time PCR in virology. Nucleic Acids Res..

[14] Schmittgen T.D., Livak K.J. (2008). Analyzing real-time PCR data by the comparative CT method. Nat. Protoc..

[15] Bustin S.A., Benes V., Garson J.A., Hellemans J., Huggett J., Kubista M., Mueller R., Nolan T., Pfaffl M.W., Shipley G.L. (2009). The MIQE Guidelines: Minimum Information for Publication of Quantitative Real-Time PCR Experiments. Clin. Chem..

[16] Elnifro E.M., Ashshi A.M., Cooper R.J., Klapper P.E. (2000). Multiplex PCR: Optimization and Application in Diagnostic Virology. Clin. Microbiol. Rev..

[17] Bernard P.S., Wittwer C.T. (2002). Real-Time PCR Technology for Cancer Diagnostics. Clin. Chem..

[18] Cherpillod P., Schibler M., Vieille G., Cordey S., Mamin A., Vetter P., Kaiser L. (2016). Ebola virus disease diagnosis by real-time RT-PCR: A comparative study of 11 different procedures. J. Clin. Virol..

[19] Dadgar N., Gonzalez-Suarez A.M., Fattahi P., Hou X., Weroha J.S., Gaspar-Maia A., Stybayeva G., Revzin A. (2020). A microfluidic platform for cultivating ovarian cancer spheroids and testing their responses to chemotherapies. Microsyst. Nanoeng..

[20] Huggett J.F., French D., O’Sullivan D.M., Moran-Gilad J., Zumla A. (2022). Monkeypox: Another test for PCR. Eurosurveillance.

[21] Xiao A.T., Tong Y.X., Zhang S. (2020). Profile of RT-PCR for SARS-CoV-2: A Preliminary Study From 56 COVID-19 Patients. Clin. Infect. Dis..

[22] Njiru Z.K., Constantine C.C., Ndung’u J.M., Robertson I., Okaye S., Thompson R.C.A., Reid S.A. (2004). Detection of Trypanosoma evansi in camels using PCR and CATT/T. evansi tests in Kenya. Vet. Parasitol..

[23] Cocolin L., Rajkovic A., Rantsiou K., Uyttendaele M. (2011). The challenge of merging food safety diagnostic needs with quantitative PCR platforms. Trends Food Sci. Technol..

[24] Liao C.-S., Lee G.-B., Wu J.-J., Chang C.-C., Hsieh T.-M., Huang F.-C., Luo C.-H. (2005). Micromachined polymerase chain reaction system for multiple DNA amplification of upper respiratory tract infectious diseases. Biosens. Bioelectron..

[25] Wilding P., Shoffner M.A., Kricka L.J. (1994). PCR in a silicon microstructure. Clin. Chem..

[26] Kopp M.U., Mello A.J., Manz A. (1998). Chemical amplification: Continuous-flow PCR on a chip. Science.

[27] Ahrberg C.D., Manz A., Chung B.G. (2016). Polymerase chain reaction in microfluidic devices. Lab A Chip.

[28] Podbiel D., Laermer F., Zengerle R., Hoffmann J. (2020). Fusing MEMS technology with lab-on-chip: Nanoliter-scale silicon microcavity arrays for digital DNA quantification and multiplex testing. Microsyst. Nanoeng..

[29] Zec H.C., Zheng T., Liu L., Hsieh K., Rane T.D., Pederson T., Wang T.-H. (2018). Programmable microfluidic genotyping of plant DNA samples for marker-assisted selection. Microsyst. Nanoeng..

[30] Basiri A., Heidari A., Nadi M.F., Fallahy M.T.P., Nezamabadi S.S., Sedighi M., Saghazadeh A., Rezaei N. (2021). Microfluidic devices for detection of RNA viruses. Rev. Med. Virol..

[31] Chen W., Luo H., Zeng L., Pan Y., Parr J.B., Jiang Y., Cunningham C.H., Hawley K.L., Radolf J.D., Ke W. (2022). A suite of PCR-LwCas13a assays for detection and genotyping of Treponema pallidum in clinical samples. Nat. Commun..

[32] Kim J., Byun D., Mauk M.G., Bau H.H. (2009). A disposable, self-contained PCR chip. Lab Chip.

[33] Zhu Q., Gao Y., Yu B., Ren H., Qiu L., Han S., Jin W., Jin Q., Mu Y. (2012). Self-priming compartmentalization digital LAMP for point-of-care. Lab A Chip.

[34] Nguyen P.Q.M., Wang M., Maria N.A., Li A.Y., Tan H.Y., Xiong G.M., Tan M.-K.M., Bhagat A.A.S., Ong C.W.M., Lim C.T. (2022). Modular micro-PCR system for the onsite rapid diagnosis of COVID-19. Microsyst. Nanoeng..

[35] Shi X., Lin L.-I., Chen S.-Y., Chao S.-H., Zhang W., Meldrum D.R. (2011). Real-time PCR of single bacterial cells on an array of adhering droplets. Lab A Chip.

[36] Oblath E.A., Henley W.H., Alarie J.P., Ramsey J.M. (2013). A microfluidic chip integrating DNA extraction and real-time PCR for the detection of bacteria in saliva. Lab A Chip.

[37] Wu J., Kodzius R., Xiao K., Qin J., Wen W. (2012). Fast detection of genetic information by an optimized PCR in an interchangeable chip. Biomed. Microdevices.

[38] Chen X., Song Q., Zhang B., Gao Y., Lou K., Liu Y., Wen W. (2021). A Rapid Digital PCR System with a Pressurized Thermal Cycler. Micromachines.

[39] Patti A., Acierno D. (2020). Thermal Conductivity of Polypropylene-Based Materials.

[40] Kaufman J. (2016). Properties and Characteristics of Aluminum and Aluminum Alloys.

[41] Mišković-Stanković V.B., Stanić M.R., Dražić D.M. (1999). Corrosion protection of aluminium by a cataphoretic epoxy coating. Prog. Org. Coat..

[42] Van den Brand J., Van Gils S., Beentjes P.C.J., Terryn H., Sivel V., de Wit J.H.W. (2004). Improving the adhesion between epoxy coatings and aluminium substrates. Prog. Org. Coat..

[43] Cheng Z., Gu Y., Li S., Wang Y., Chen H., Cheng J., Liu P. (2017). Enclosed casting of epoxy resin for rapid fabrication of rigid microfluidic chips. Sens. Actuators B Chem..

[44] Sobhani S., Jannesari A., Bastani S. (2012). Effect of molecular weight and content of PDMS on morphology and properties of silicone-modified epoxy resin. J. Appl. Polym. Sci..

[45] Ng W., Seet H., Lee K., Ning N., Tai W., Sutedja M., Fuh J., Li X. (2009). Micro-spike EEG electrode and the vacuum-casting technology for mass production. J. Mater. Process. Technol..

[46] Patel N. (2020). Study on computer numerical control (CNC) technology. Int. Res. J. Eng. Technol. (IRJET).

[47] Mamadjanov A.M., Yusupov S.M., Sadirov S. (2021). Advantages and the future of cnc machines. Sci. Prog..

[48] Song Q., Sun X., Dai Z., Gao Y., Gong X., Zhou B., Wu J., Wen W. (2021). Point-of-care testing detection methods for COVID-19. Lab A Chip.

[49] Cozma A., Jakobsen H., Tilli M., Paulasto-Krockel M., Petzold M., Theuss H., Motooka T., Lindroos V. (2020). Chapter 24-Anodic bonding. Handbook of Silicon Based MEMS Materials and Technologies.

[50] Vijayan P.P., Formela K., Saeb M.R., Chithra P.G., Thomas S. (2022). Integration of antifouling properties into epoxy coatings: A review. J. Coat. Technol. Res..

[51] Kodzius R., Xiao K., Wu J., Yi X., Gong X., Foulds I.G., Wen W. (2012). Inhibitory effect of common microfluidic materials on PCR outcome. Sens. Actuators B Chem..

[52] Zhou C., Li R., Luo W., Chen Y., Zou H., Liang M., Li Y. (2016). The preparation and properties study of polydimethylsiloxane-based coatings modified by epoxy resin. J. Polym. Res..

[53] Vilčáková J., Kutějová L., Jurča M., Moučka R., Vícha R., Sedlačík M., Kovalcik A., Machovský M., Kazantseva N. (2018). Enhanced Charpy impact strength of epoxy resin modified with vinyl-terminated polydimethylsiloxane. J. Appl. Polym. Sci..

[54] Nishimoto S.K., Nishimoto M., Park S.-W., Lee K.-M., Kim H.-S., Koh J.-T., Ong J.L., Liu Y., Yang Y. (2008). The effect of titanium surface roughening on protein absorption, cell attachment, and cell spreading. Int. J. Oral Maxillofac. Implant..

[55] Tong R., Zhang L., Song Q., Hu C., Chen X., Lou K., Gong X., Gao Y., Wen W. (2019). A fully portable microchip real-time polymerase chain reaction for rapid detection of pathogen. Electrophoresis.

[56] Zhang B., Liu Y., Song Q., Li B., Chen X., Luo X., Wen W. (2022). A new dynamic deep learning noise elimination method for chip-based real-time PCR. Anal. Bioanal. Chem..

[57] Akbari M., Doré Hansen M., Halgunset J., Skorpen F., Krokan H.E. (2005). Low Copy Number DNA Template Can Render Polymerase Chain Reaction Error Prone in a Sequence-Dependent Manner. J. Mol. Diagn..

[58] Chen X., Liu Y., Zhan X., Gao Y., Sun Z., Wen W., Zheng W. (2022). Ultrafast PCR Detection of COVID-19 by Using a Microfluidic Chip-Based System. Bioengineering.

[59] Yang S., Wen W. (2021). Lyophilized Ready-to-Use Mix for the Real-Time Polymerase Chain Reaction Diagnosis. ACS Applied Bio Materials.

