# Supplementary material for: An Aluminum-Based Microfluidic Chip for Polymerase Chain Reaction Diagnosis
Source: Molecules. 2023 Jan 21;28(3):1085. doi: 10.3390/molecules28031085 (PMC9921548; doi:10.3390/molecules28031085)
Supplement: Supplementary file 1 [file molecules-28-01085-s001.zip › molecules-2155191-supplementary.pdf]

An Aluminum-based Microfluidic Chip for Polymerase Chain Reaction Diagnosis

Siyu Yang [1], Ziyi Zhang [1], Qingyue Xian [1], Qi Song [2], Yiteng Liu [1], Yibo Gao [2], and Weijia Wen [2,3,4,*]

Supplementary Materials

Table S1. Information of primers and probes used in this study.

Primers and probes	Sequences (5′ to 3′)
C-HBV2 Pb478	6-FAM-ACTCCCTCGCCTCGCAGACGA-BHQ-1
C-HBV2 F382	ACTCCTCCAGCTTATAGACCACCA
C-HBV2 R518	CGACGCGGCGATTGAGA

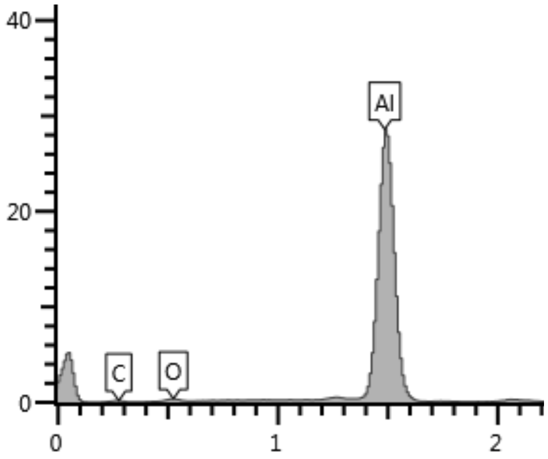

Figure S1. EDS map sum spectrum for the surface of chips before coating with silicone-modified epoxy resin.

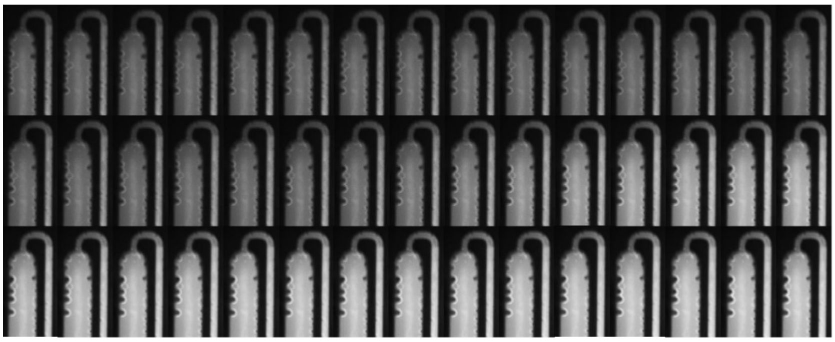

Figure S2. Fluorescent images from each cycle of the test in the aluminum-based chip.
